# Maternal Supply of Cas9 to Zygotes Facilitates the Efficient Generation of Site-Specific Mutant Mouse Models

**DOI:** 10.1371/journal.pone.0169887

**Published:** 2017-01-12

**Authors:** Alberto Cebrian-Serrano, Shijun Zha, Lars Hanssen, Daniel Biggs, Christopher Preece, Benjamin Davies

**Affiliations:** 1 Wellcome Trust Centre for Human Genetics, University of Oxford, Oxford, United Kingdom; 2 MRC Weatherall Institute of Molecular Medicine, University of Oxford, Oxford, United Kingdom; Osaka University, JAPAN

## Abstract

Genome manipulation in the mouse via microinjection of CRISPR/Cas9 site-specific nucleases has allowed the production time for genetically modified mouse models to be significantly reduced. Successful genome manipulation in the mouse has already been reported using Cas9 supplied by microinjection of a DNA construct, *in vitro* transcribed mRNA and recombinant protein. Recently the use of transgenic strains of mice overexpressing Cas9 has been shown to facilitate site-specific mutagenesis via maternal supply to zygotes and this route may provide an alternative to exogenous supply. We have investigated the feasibility of supplying Cas9 genetically in more detail and for this purpose we report the generation of a transgenic mice which overexpress Cas9 ubiquitously, via a CAG-Cas9 transgene targeted to the *Gt(ROSA26)Sor* locus. We show that zygotes prepared from female mice harbouring this transgene are sufficiently loaded with maternally contributed Cas9 for efficient production of embryos and mice harbouring indel, genomic deletion and knock-in alleles by microinjection of guide RNAs and templates alone. We compare the mutagenesis rates and efficacy of mutagenesis using this genetic supply with exogenous Cas9 supply by either mRNA or protein microinjection. In general, we report increased generation rates of knock-in alleles and show that the levels of mutagenesis at certain genome target sites are significantly higher and more consistent when Cas9 is supplied genetically relative to exogenous supply.

## Introduction

Recent progress in the application of CRISPR/Cas9 nucleases has transformed our ability to manipulate the genome site-specifically [1–3]. These targeted nucleases allow precise and efficient genome editing of model organisms by direct injection of the CRISPR/Cas9 components into the one-cell stage embryo, bypassing the need for embryonic stem cells and accelerating the process of genome modification[4].

The CRISPR/Cas9 technology, originally derived from the type II adaptive immune system of *Streptococcus pyogenes*, has been modified into a two-component system for mutagenesis in the laboratory[5, 6]. Cas9, the nuclease, is active when it forms a complex with a single guide-RNA (sgRNA) molecule, which represents a chimeric fusion of two RNA species, the tracrRNA and the crRNA. The first 20 nucleotides of the crRNA sequence define the specificity of the nuclease, which occurs by complementary base pairing with the target sequence within genomic DNA. The activated nuclease generates a double-strand break (DSB) at the target site, which is readily repaired by the cell’s DNA repair machinery. Repair by non-homologous end joining (NHEJ) can introduce indel mutations at the DSB site[7], providing a means of disrupting protein coding or other functional DNA sequences. An alternative pathway, homology directed repair (HDR) can be adopted by the cell to repair the lesion using homologous sequences as a template. The HDR pathway can be employed to introduce defined mutations into the genome by presenting the cell with a homologous template harbouring desired nucleotide changes[6]. Where small changes are required, this can be achieved with a single stranded oligodeoxynucleotide (ssODN) template[8].

Lines of genetically modified mice can now be generated using CRISPR/Cas9 site-specific nucleases at unprecedented speed. Conventionally this is achieved by microinjection of fertilized oocytes with sgRNA (or its constituent tracrRNA and crRNA) together with Cas9 provided as either a DNA construct[9], an mRNA[10] or protein[11], and homology directed repair (HDR) templates, where required[12].

Despite all the recent advances, there are still important limitations of the CRISPR/Cas9 system with respect to mouse model development and further optimization and simplification would be welcomed. Firstly, variations in the quality of the Cas9 mRNA or protein microinjected contribute significantly to variation in experimental outcome and a more standardized way of dosing Cas9 within the embryo would be advantageous. Secondly, CRISPR/Cas9-mediated mutagenesis by zygote microinjection frequently leads to mosaicism and allele complexity in founders[13]. This occurs when the CRISPR/Cas9 machinery remains active within the embryo after the first cleavage event and generates different mutations in the daughter cells. Individual founder mice may show germline mosaicism, and thus transmit unexpected mutations, complicating the downstream breeding experiments and increasing animal and financial cost. Lastly, NHEJ is favoured over HDR as the repair pathway for induced DSBs, which can make the introduction of directed changes for knock-in mouse model production challenging.

As an alternative to exogenous Cas9 supply and providing possible solutions for the above, genetic supply of Cas9 has been explored using transgenic lines overexpressing Cas9. Efficient somatic mutagenesis has been demonstrated by the delivery of sgRNAs alone into tissue and cells of mice overexpressing Cas9[14, 15], proving genetic Cas9 supply can mediate mutagenesis and demonstrating that ubiquitous expression of Cas9 in mice harbours no apparent detrimental effects. Recent reports have also demonstrated that microinjection of sgRNAs into mouse zygotes derived from transgenic mice expressing Cas9 both ubiquitously[16] and under the control of an oocyte specific promoter[17] results in site-specific mutagenesis, confirming that genetically supplied Cas9 is active in the early embryo. Indeed, mutagenesis was seen in embryos that had not inherited the Cas9 transgene, implying that the active nuclease is contributed maternally.

Maternally contributed Cas9 within the zygote would provide early and transient nuclease activity, which may influence allele complexity and levels of mutagenesis when compared with exogenous supply. The type of the supply has previously been shown to influence the timing of nuclease action: Cas9 delivered as a protein has been shown to have a faster action in cell lines[18] and zebrafish embryos[11] when compared to delivery as plasmid or mRNA. Interestingly, immediate and transient nuclease action has been considered advantageous for homology directed repair (HDR) events following CRISPR/Cas9 genome cleavage. Indeed, a tendency of increased knock-in efficiency when compared to exogenous supply of Cas9 mRNA has been shown in certain contexts in microinjection studies in mice[19, 20], rats[20] and zebrafish[21], potentially as the co-microinjected donor template can participate in the repair mechanism immediately upon DNA cleavage.

In this study, we aim to explore the genetic supply of Cas9 for the generation of mouse models in more detail, extending the repertoire of alleles that can be generated to point mutation Knock-in alleles and examine the efficiency of their generation compared to exogenous Cas9 supply. We present a targeted-transgenic Cas9 overexpression mouse model and demonstrate that maternally contributed Cas9 within the early embryo is sufficient to achieve high levels of site-specific mutagenesis and efficient production of mutant founders and lines, harbouring point mutation knock-in, loss-of-function indel and genomic deletion alleles. We include a side-by-side comparison of mutagenesis efficiencies following Cas9 delivery as mRNA, protein or as genetic supply using our Cas9 overexpression model and provide evidence that genetic Cas9 can increase the efficiency of mutant mouse production.

## Materials and Methods

### Generation of Cas9 overexpressing mice

A cDNA encoding NLS-Cas9 was isolated from pX330 (Addgene #42230) by EcoRI digestion and cloned upstream of the ubiquitous CAG promoter within a PhiC31 integrase mediated cassette exchange vector [22]. 5 μg of this exchange plasmid together with 5 μg of a PhiC31 expression plasmid was electroporated into 1×10^6^ IDG26.10–3 ES cells using the Neon transfection system (Life Technologies) (3×1400 V, 10 ms), selected in 210 μg/ml G418, and screened for targeted integration at the *Gt(ROSA26)Sor* locus as previously described [23]. Targeted embryonic stem cells were microinjected into C57BL/6J blastocysts and the resulting chimeras were mated with C57BL/6J females to establish a line of targeted transgenic mice overexpressing NLS-Cas9. Mice were backcrossed for at least 3 generations with C57BL/6J prior to intercrossing to generate homozygous mice or for use in microinjection experiments.

### Validation of genome editing using a genetic supply of Cas9 in embryonic fibroblasts

Complimentary oligonucleotides containing the sgRNA target sequences for *Trp53* and *Cdkn2a* (Table A in S1 File) were annealed and cloned into the BsaI site of a plasmid containing a human U6 promoter and the invariant part of the mature sgRNA sequence. Mouse embryonic fibroblasts were isolated from heterozygous Cas9 expressing and wild-type mid-gestation embryos and transfected with these two plasmids. Cultures were passaged at confluency for 6 passages and then plated at low density (1000 cells per 10 cm dish) and the resulting colonies stained with 0.01% crystal violet. Genomic DNA was prepared from the remaining cells and genotyped by Sanger sequencing of PCR products amplifying the target sites within the *Trp53* and the *Cdkn2a* genes (Table A in S1 File).

### Cloning of guide-RNAs and preparation of RNAs for microinjection

Complimentary oligonucleotides containing the sgRNA target sequences (Table B in S1 File) were annealed and cloned into the BbsI site of pX330 (Addgene #42230) as previously described [24]. DNA templates for *in vitro* transcription were generated from these plasmids using forward primers with a 5’ extension corresponding to a T7 polymerase binding site in combination with a reverse primer, gRNA-R (5’-AAAAGCACCGACTCGGTGCC-3), binding downstream of the mature sgRNA sequence (Table B in S1 File). RNAs were prepared by *in vitro* transcription using the MEGAshortscript™ T7 Transcription Kit (ThermoFisher Scientific). Capped mRNA for NLS-Cas9 was generated by firstly cloning the NLS-Cas9 cDNA from pX330 into pcDNA3.1, linearizing the plasmid with XhoI and using this as a template for *in vitro* transcription using the mMESSAGE mMACHINE® T7 Ultra Kit (ThermoFisher Scientific). *In vitro* transcribed RNAs were purified using the MEGAclear Kit (ThermoFisher Scientific) and diluted prior to microinjection in 10 mM Tris.HCl pH7.5, 0.1 mM EDTA pH8.0. A 69mer tracrRNA and a 42mer crRNA (GUUACUUACCCAAGGCAUGCGUUUUAGAGCUAUGCUGUUUUG) were synthesized (Sigma-Aldrich).

### Zygote microinjection and mouse production

Female mice, homozygous, heterozygous or wild-type for the CAG-Cas9 transgene insertion at 3 weeks of age were superovulated and mated with wild-type C57BL/6J studs. Fertilized oocytes were prepared from plugged females and microinjected into a pronucleus with either 20 ng/μl of sgRNA (or tracrRNA:crRNA) (for oocytes derived from transgenic CAG-Cas9 females) or 20 ng/μl sgRNA and 10 ng/μl NLS-Cas9 mRNA or 100 ng/ul recombinant NLS-Cas9 protein (PNA Bio Inc.) (for zygotes derived from wild-type matings). Where required, ssODN templates (Eurogentec) for homology directed repair (Table D in S1 File) were added to the microinjection mix at a final concentration of 20 ng/μl. Microinjected zygotes were cultured overnight to the two-cell stage and surgically implanted into recipient CD1 females, or were cultured for a further 72 hours until blastocyst stage.

### Genotyping and quantification of mutagenesis within embryos

*In vitro* cultured blastocysts or ear biopsies from the resulting mice were lysed using standard conditions and genomic DNA purified. The target gene was amplified using the primers listed in Table B in S1 File and Sanger sequenced to establish the nature of the mutation. Where ambiguous mixed traces were obtained, the PCR product was cloned into pCR2.1-TOPO (ThermoFisher Scientific) and multiple plasmids sequenced to establish the identity of the individual alleles present. Sanger sequence traces obtained from genomic DNA prepared from whole embryos and mice was analysed using the TIDE algorithm [25] (https://tide.nki.nl/). Alleles were counted using *p* value significance cut-off of *p*<0.001.

### Assessment of off-target site within individual founder mice

The six most likely off-target sites based on mismatch prediction of the sgRNA 5’-CCACAGTCAGGAAAGCAGCA-3’ targeting chrX(-):41620875–41620894 were established using the crispr.mit.edu algorithm which ranks putative off-targets using experimentally-determined effects of mismatched positions. Three founder mice harbouring indel mutations at the target site, were genotyped at these six loci using specific PCRs (Table F in S1 File) followed by Sanger sequencing to assess mutagenesis.

### Animal work

All animal studies received ethical approval from the Clinical Medicine AWERB (Animal Welfare and Ethical Review Body) at the University of Oxford and were performed in accordance with UK Home Office Animals (Scientific Procedures) Act 1986 under project license PPL 30/3085. Mice were housed in individually ventilated cages and received food and water ad libitum. All surgery was performed under isoflurane inhalation anaesthesia using appropriate analgesia, and all efforts were made to minimize suffering.

### Statistical analysis

Results of mutant pups per total pups born, 2-cell progression, live birth rates, percentages of mutant blastocysts and HDR-mediated knock-in rates were analysed using the chi-square test. Percentage of mutagenic events at the DNA level, number of alleles in targeted embryos and litter size differences among groups were analysed by one-way ANOVA. *p*<0.05 was considered statistically significant.

## Results

### Generation and *in vitro* validation of Cas9 overexpressing mice

**Fig 1:**
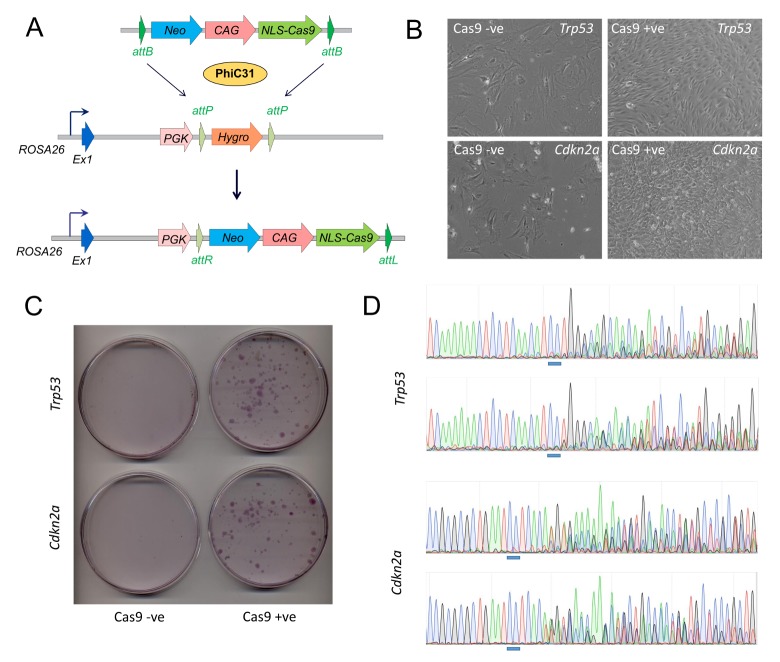
Generation and validation of Cas9 overexpressing mice. A) PhiC31 integrase mediated cassette exchange allows the transgenic targeting of a CAG-NLS-Cas9 overexpression cassette into the *ROSA26* locus (*Gt(ROSA26)Sor*) in embryonic stem cells. B) Primary embryonic fibroblast cultures grown from Cas9 expressing (Cas9 +ve) or wild-type littermate embryos (Cas9 -ve) and transfected with sgRNAs targeted to exon 1 of the tumor suppressor genes *Trp53* and *Cdkn2a*. Clusters of cell foci with an immortalized appearance are only evident in Cas9 expressing cultures, consistent with a loss-of-function of these two genes. C) Clonogenic assay of Cas9 expressing and wild-type embryonic fibroblast cultures following transfection with guide-RNAs targeted to *Trp53* and *Cdkn2a*. Clones of cells are only seen in Cas9 expressing cultures, indicating an immortalized phenotype consistent with loss-of-function of these two genes. D) Sanger sequence traces from amplicons generated from pooled cells at the *Trp53* and *Cdkn2a* loci in two independent Cas9 expressing fibroblast cultures transfected with sgRNAs targeted to *Trp53* and *Cdkn2a*. The appearance of mixed sequences traces appearing near the protospacer-adjacent-motif (blue bar) are indicative of multiple indel mutations at these target sites.

We generated a targeted transgenic mouse model overexpressing Cas9 ubiquitously by integrating a CAG-Cas9 construct at the *Gt(ROSA26)Sor* locus via PhiC31 integrase cassette exchange in embryonic stem cells (Fig 1A) [22]. This same strategy was adopted for the Cas9 overexpression mice generated by Platt et al.[14] and more recently by Chu et al.[15], where investigation revealed no obvious phenotypic consequence of Cas9 overexpression in these mice. Accordingly, heterozygous or homozygous mice generated in this study revealed no overt phenotype and were behaviourally indistinguishable from their wild-type littermates. All genotypes were fully fertile and no significant difference in average litter size between groups of breeding homozygous females and wild-type females was found (homozygous female: 6.8±0.9, n = 21; wild-type female: 6.6±0.6, n = 19; *p*>0.05).

We verified that genetic Cas9 supply in our model could mediate genome engineering by analysing embryonic fibroblasts derived from Cas9 expressing embryos. Transfection of Cas9 expressing fibroblasts with sgRNAs targeted against the tumor suppressor genes, p53 (*Trp53*) and p14 (*Cdkn2a*) lead to the expected immortalization phenotype consistent with loss-of-function of these genes as evidenced by cell morphology (Fig 1B) and efficient colony formation upon low density plating (Fig 1C). In contrast, transfection of embryonic fibroblasts derived from wild-type litter mates with the above sgRNAs resulted in no immortalization and, after a few passages, the majority of cells had senesced (Fig 1B) and cells were unable to form colonies following low density plating (Fig 1C). Genotyping by Sanger sequence confirmed that both genes were inactivated by indel mutation at the target sites only in the transfected Cas9 expressing fibroblasts (Fig 1D). The CAG-Cas9 transgene expression is thus sufficient for the genetic supply of Cas9 enabling site-specific indel mutation.

### Cas9 mediating genome editing in zygotes is maternally contributed from Cas9 overexpressing mice

**Fig 2:**
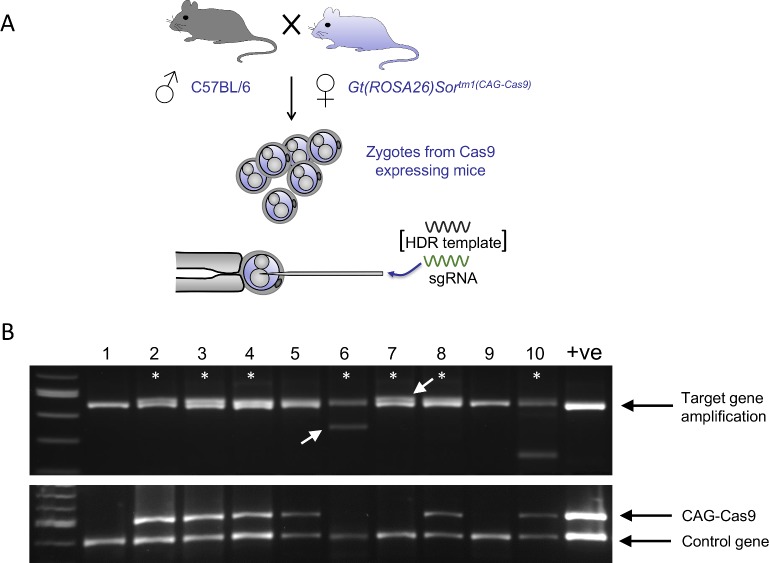
Maternal supply of Cas9 within fertilized zygotes is sufficient for efficient genome editing. A) Zygotes are obtained from matings between C57BL/6J stud males and heterozygous female mice, constitutively overexpressing Cas9. Microinjection of the resulting pronuclear stage zygotes was performed using sgRNA alone. B) Top panel shows target site amplification in blastocysts after microinjection of fertilized zygotes generated from heterozygous Cas9 expressing females with sgRNA against target chr17(+):34030557–34030576. Doublets, indicated by asterisks, are indicative of indel mutagenesis. Bottom panel shows the same blastocysts genotyped for the presence of the Cas9 transgene and a control gene. Genetically wild-type embryos (6 and 7) show clear mutagenesis (white arrows), proving that maternally contributed Cas9 is sufficient for genome editing.

We next investigated whether our mouse model, which harbours only a single copy of the Cas9 overexpression construct within the *Gt(ROSA26)Sor* locus, was able to supply sufficient Cas9 for genome engineering within the fertilized zygote (Fig 2A). Previous studies microinjecting both mouse[16] and drosophila[26, 27] embryos derived from Cas9 overexpressing mothers indicated that the Cas9 responsible for the mutagenesis was contributed as maternal transcripts or protein laid down in the developing oocyte. To formally prove that this was also the case with our transgenic targeted line of Cas9 expressing mice, we mated female mice, heterozygous for the CAG-Cas9 transgene, with wild-type studs and microinjected the resulting zygotes with a specific sgRNA targeting protospacer chr17(+):34030557–34030576) (Table B in S1 File). Assuming Mendelian inheritance of the CAG-Cas9 transgene, only half of the zygotes would be expected to harbour the transgene.

Following microinjection, the embryos were cultured until the blastocyst-stage, genomic DNA was prepared and each whole embryo was genotyped for mutagenesis at the CRISPR/Cas9 cleavage site and for the presence or absence of the CAG-Cas9 transgene, along with amplification of a control gene (Table C in S1 File). Multiple embryos (70% of embryos analyzed) were identified which harboured indel mutations at the target gene as evidenced by polymorphic amplification products on a high percentage agarose gel (Fig 2B upper panel), thus confirming that genetically supplied Cas9 from our targeted transgene is both active within the pre-implantation embryo and able to mediate genome engineering. Furthermore, successful mutagenesis in the absence of inheritance of the CAG-Cas9 transgene was observed (Fig 2B lower panel), confirming that the Cas9 contributed to the oocyte by the transgenic mother was the source of the Cas9 nuclease responsible for the mutagenesis.

In agreement, when zygotes were derived from mating transgenic Cas9-expressing male mice with wild-type females and used for microinjection with sgRNA alone, no significant mutagenesis at the genomic target site was observed (from 9 embryos sequenced there was no evidence of indel mutation by Sanger sequencing). Thus, embryonic expression of the integrated Cas9 transgene was presumably negligible in the early pre-implantation embryos.

### Quantitative, whole-embryo analysis of genetic Cas9-supply versus exogenous supply by mRNA and protein for indel mutagenesis

Having demonstrated that maternal supply of Cas9 from overexpressing mothers was suitable for efficient genome engineering, we performed a more quantitative analysis of mutagenesis by genotyping the complete embryo and examining the mutation rate and the absolute levels of mutagenesis occurring within each mutant embryo. Zygotes derived from either homozygous or heterozygous Cas9 expressing mothers were microinjected with two different sgRNAs targeting protospacers chr17(+):34030557–34030576 and chr5(-):123582755–123582774 (Table B in S1 File) and, in parallel, wild-type zygotes were microinjected with the same sgRNAs together with exogenous Cas9 mRNA or Cas9 protein. Microinjected embryos were cultured to the blastocyst stage, lysed and the target sequences within the complete embryo were amplified by PCR and mutagenesis assessed by Sanger sequencing and quantified by TIDE analysis[25].

**Fig 3:**
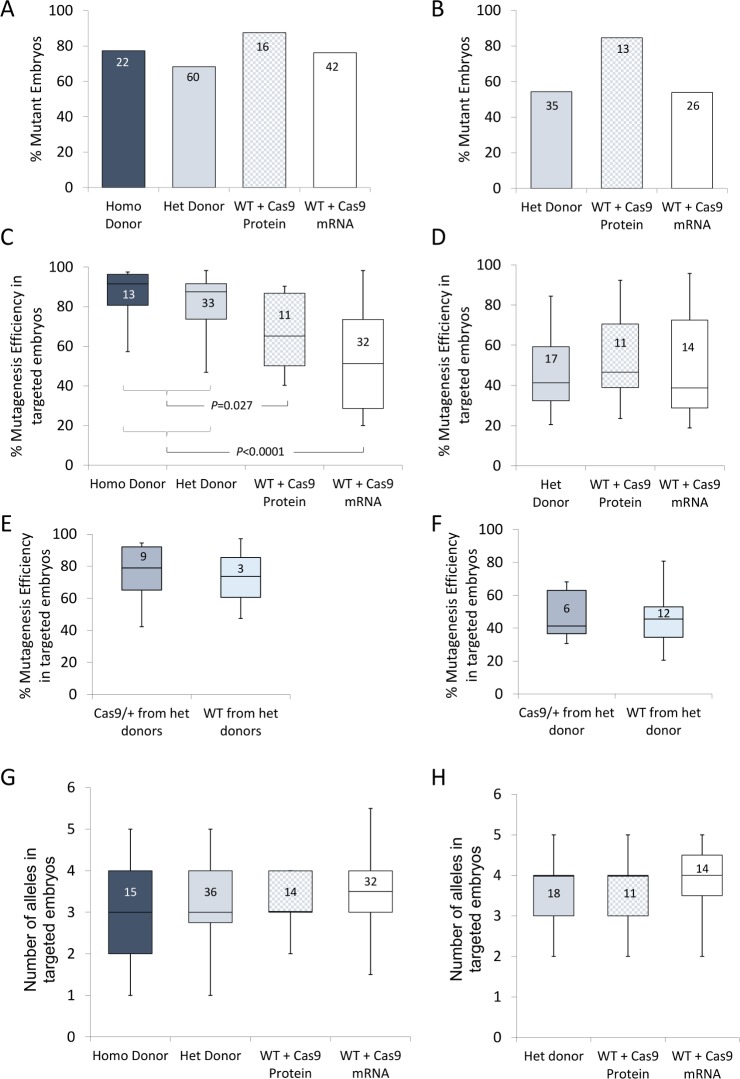
Comparison of gene editing with maternal supply of Cas9 with Cas9 mRNA or protein injection. Data in panels A, C, E, G from microinjection of sgRNA chr17(+):34030557–34030576 and panels B, D, F, H for sgRNA chr5(-):123582755-123582774.A) and B) Percentage mutation rates (mutant embryos per total number) for maternal Cas9 supply (homozygous donor and/or heterozygous donor) and exogenous Cas9 supply as mRNA or protein (wild-type donor) supply, showing similar efficiencies. C) and D) Box plot showing the range of mutagenesis (amount of wild-type sequence mutation within a single mutant blastocyst) for maternal Cas9 supply and exogenous Cas9 supply as mRNA and protein (wild-type donor). The ends of the whiskers are at 1.5 interquartile ranges above/below the third/first quartiles. E) and F) As for C) and D) but comparing mutagenesis efficiency in either heterozygous (Cas9/+) or wild-type (WT) embryos derived from microinjected zygotes obtained from heterozygous Cas9 expressing females. E) and F) Box blot showing the range of alleles identified in mutant embryos generated from maternal Cas9 supply and exogenous supply as mRNA and protein. Data inside the bars are the number of embryos sampled.

We found no significant difference in the rate of mutagenesis between zygotes supplied genetically with Cas9 and wild-type embryos supplied with exogenous Cas9 as either mRNA or protein (Fig 3A and 3B). Furthermore, mutagenesis rates were similar in microinjected embryos derived from zygotes obtained from homozygous and heterozygous donors (Fig 3A).

When we examined the degree of mutagenesis in each mutant embryo, significant differences were observed between genetic supply of Cas9 and exogenous supply by Cas9 mRNA or protein microinjection, with one of the sgRNAs tested. TIDE analysis[25] was performed on the amplicons obtained from the whole blastocysts, providing an estimation of the percentage of mutated sequences in a particular individual. In the experiments using sgRNA targeting chr17(+):34030557–34030576, we found the absolute level of mutagenesis was significantly increased within each mutant embryo when Cas9 had been genetically supplied, compared to exogenous supply as mRNA (*p<*0.0001) and protein (*p<*0.05) (Fig 3C). Furthermore, the spread of data was very different, with zygotes receiving Cas9 exogenously via mRNA microinjection showing a much larger variation in mutagenic outcome. In contrast, genetic supply from both homozygous and heterozygous Cas9 overexpressing female mice, which showed no significant difference between these genotypes, revealed lower variation in the extent of mutagenesis. Interestingly, zygotes receiving Cas9 via protein microinjection showed an intermediate level of variation. In contrast, when examining the data obtained from microinjection of sgRNA chr5(-):123582755–123582774, no significant difference in the degree of mutagenesis was observed between groups (Fig 3D). Overall, these data highlight the difficulties of drawing conclusions based on a single genomic locus but do suggest that, at least at certain loci, genetic supply of Cas9 can result in higher and more consistent levels of nuclease activity within the microinjected zygote.

A more precise analysis of microinjected zygotes obtained from heterozygous donors allowed an assessment of mutational efficacy in embryos that had inherited the CAG-Cas9 transgene and embryos that had not. No significant difference between transgenic and wild-type zygotes were observed (Fig 3E and 3F), consistent with the observation that it is maternally contributed Cas9 (rather than embryonic expressed Cas9) that is predominantly responsible for the nuclease activity mediating the genome engineering events.

TIDE analysis also allows an assessment of the predominant alleles found in the mutant whole embryos and thus provides a measure of the degree of mosaicism present following microinjection of the CRISPR/Cas9 nucleases. Detection of more than two alleles in an embryo is indicative of mosaicism and thus we were interested to explore whether the genetic supply of Cas9 had any influence on allele complexity. Using the two sgRNAs, we found no significant association between the number of alleles and the mode of Cas9 supply, genetic, mRNA or protein (Fig 3G and 3H), with all groups showing a degree of mosaicism.

### Quantitative, whole-embryo analysis of knock-in efficiencies with genetic Cas9-supply versus exogenous supply by mRNA

**Fig 4:**
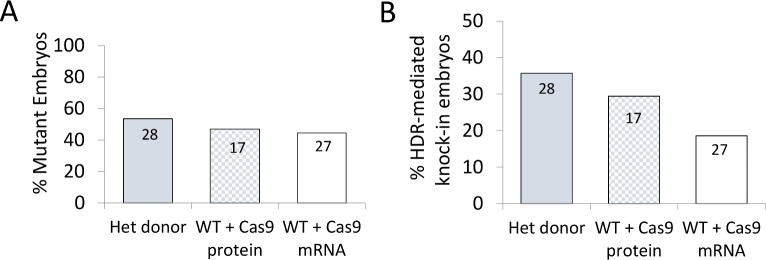
Comparison of maternal supply of Cas9 with exogenous Cas9 mRNA injection for the production of knock-in alleles. A) Percentage mutation rates (mutant embryos per total number) for maternal Cas9 supply (heterozygous donor) and exogenous Cas9 supply as mRNA or protein (wild-type donor). B) Percentage of total embryos harbouring the desired point mutation knock-in allele for maternal Cas9 supply and exogenous Cas9 supply as mRNA or protein. Data inside the bars are the number of embryos sampled.

Previous reports using genetic supply of Cas9 only established the feasibility of using this approach for the generation of indel mutant alleles caused by NHEJ of CRISPR/Cas9 cleaved target loci. To establish whether genetic supply was also suitable for alleles generated by HDR for the purpose of point mutation knock-in, zygotes prepared from Cas9 expressing females mated with wild-type male mice were microinjected with sgRNA targeting protospacer chr5(-):123582755–123582774 (Table B in S1 File) and an ssODN designed to introduce a point mutation change and a novel TspGWI restriction site (Table D in S1 File). In addition, to evaluate any advantage in HDR efficiency that genetic Cas9 supply may provide, the same sgRNA and ssODN template were microinjected into C57BL/6J zygotes, prepared from wild-type matings, together with Cas9 mRNA or Cas9 protein to allow a comparison with exogenous supply. The resulting microinjected embryos were cultured to the blastocyst stage, lysed and the target locus amplified and digested with TspGWI to establish the rate of knock-in, followed by confirmation with Sanger sequencing. As previously, no difference in the frequency of overall mutagenesis was found between groups (Fig 4A). However, with respect to knock-in efficiency, although no statistically significant difference was found between genetic and exogenous Cas9 supply (Chi-squared test, *p*>0.05), zygotes supplied genetically with maternal Cas9 showed a clear increase in knock-in efficiency of 36% whereas exogenous supply as mRNA yielded a knock-in efficiency of 19% (Fig 4B). Intermediate levels of knock-in efficiency (29%) were obtained when Cas9 was supplied as a protein.

### Comparison between genetic Cas9-supply and exogenous supply by mRNA and protein for the generation of mutant mice

**Fig 5:**
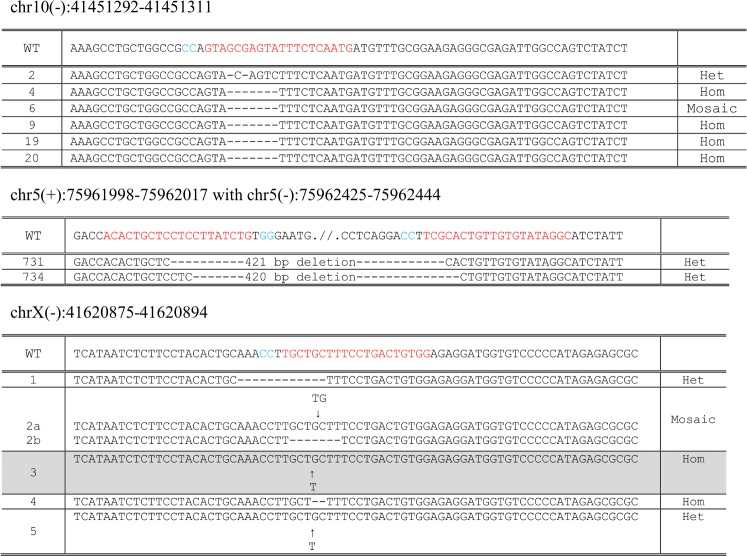
Indel and genomic deletion alleles identified in mutant pups generated from microinjection of sgRNA into fertilized zygotes derived from Cas9 expressing female mice. Alleles identified in each mutant founder mice are aligned against the wild-type sequence. In homozygous mutant mice, only one allele is shown. In heterozygous alleles with an intact wild-type allele present, only the mutant allele is shown. In compound heterozygous mutant mice with two mutant alleles, both alleles are shown. Where more than two alleles, in addition to the wild-type sequence are present (mosaic founders), the mutant alleles identified are shown. Protospacers in the wild-type sequence are shown in red, with the PAM motif highlighted in blue. Where the mutated offspring had not inherited the Cas9 transgene, the entry in the table is marked with a grey background.

The above embryo culture experiments demonstrated that genetic supply of Cas9 was able to achieve robust rates of mutagenesis in microinjected zygotes. We next explored at 3 further genomic loci whether the methodology was suitable for the generation of live mice. Female mice, homozygous and heterozygous for the Cas9 transgene, were mated with wild-type C57BL/6J studs and the resulting zygotes were microinjected with sgRNAs designed to target 3 different genes. One of experiments was performed with two sgRNA designed against target sequences in *cis* to assess the potential for genomic deletion. In parallel, the same sgRNAs were tested in combination with exogenous Cas9 as either mRNA or protein microinjected into wild-type C57BL/6J embryos to allow a comparison between genetic and exogenous supply. Microinjected zygotes were cultured overnight and transferred to foster mothers. No significant difference was seen in the behaviour of embryos derived from Cas9 overexpressing mice compared with wild-type embryos following microinjection; 2 cell progression and live birth rates were unchanged (Table E in S1 File). The resulting offspring were genotyped at the target loci by PCR amplification and Sanger sequencing to establish the number of mutant pups and to identify the predominant alleles present. Table 1 summarizes the results and the predominant alleles obtained in the resulting pups, generating with genetic Cas9 supply are shown in detail in Fig 5.

**Table 1:** Comparison of production data for different sources of Cas9.

Genomic targets (protospacers)	Cas9 Supply	Injection mix	Genotype of oocyte donor	Number of embryos transferred	Number of pups / transferred embryos (%)	Mutant pups / analyzed pups (%)
chr10(-): 41451292–41451311	Genetic	20 ng/ul gRNA	Homozygous	53	40%	6/21 (29%)
	mRNA	20 ng/ul gRNA	WT	36	17%	0/6 (0%)
		10 ng/ul Cas9 mRNA				
	Protein	20 ng/ul gRNA	WT	47	21%	1/10 (10%)
		100 ng/ul Cas9 Protein				
chr5(+): 75961998–75962017 + chr5(-): 75962425–75962444	Genetic	20 ng/ul gRNA	Homozygous	52	13%	3/7 (43%)*
	Protein	20 ng/ul gRNA	WT	92	28%	6/26 (23%)**
		100 ng/ul Cas9 Protein				
chrX(-): 41620875–41620894	Genetic	20 ng/ul gRNA	Heterozygous	12	42%	5/5 (100%)§
	mRNA	20 ng/ul gRNA	WT	68	29%	5/18 (28%)
		10 ng/ul Cas9mRNA				
	Protein	20 ng/ul gRNA	WT	30	13%	3/4 (75%)
		100 ng/ul Cas9 Protein				

* 2 of these 3 mutants carried a large deletion between the two genomic targets in cis (2/7 (29%))

** 3 of these 3 mutants carried a large deletion between the two genomic targets in cis (3/26 ((11%))

§ Mutant mice were identified which were negative for the Cas9 transgene (see Fig 5).

Robust levels of site-specific mutagenesis were seen at all 3 tested loci (mutant pups per litter: 29–100%), confirming that genetically supplied Cas9 from our targeted transgene is suitable for the generation of genetically modified mice. Interestingly, at all 3 loci, genetic supply of Cas9 yielded higher percentages of mutant pups when compared with the rates obtained using exogenous supply by both mRNA and protein, with this difference reaching statistical significance for sgRNA chrX(-):41620875–41620894 (*p*<0.01). As expected, the microinjection of 2 sgRNAs in *cis*, led to the efficient generation of genomic deletion alleles with genetic supply (29%, 2 out of 7 pups). The two founder mice generated from this experiment were both found to transmit the deletion allele, confirming that genetic supply of Cas9 is also suitable for the generation of lines of mutant mice.

### Genetic Cas9 supply allows efficient generation of point mutation knock-in mice

**Fig 6:**
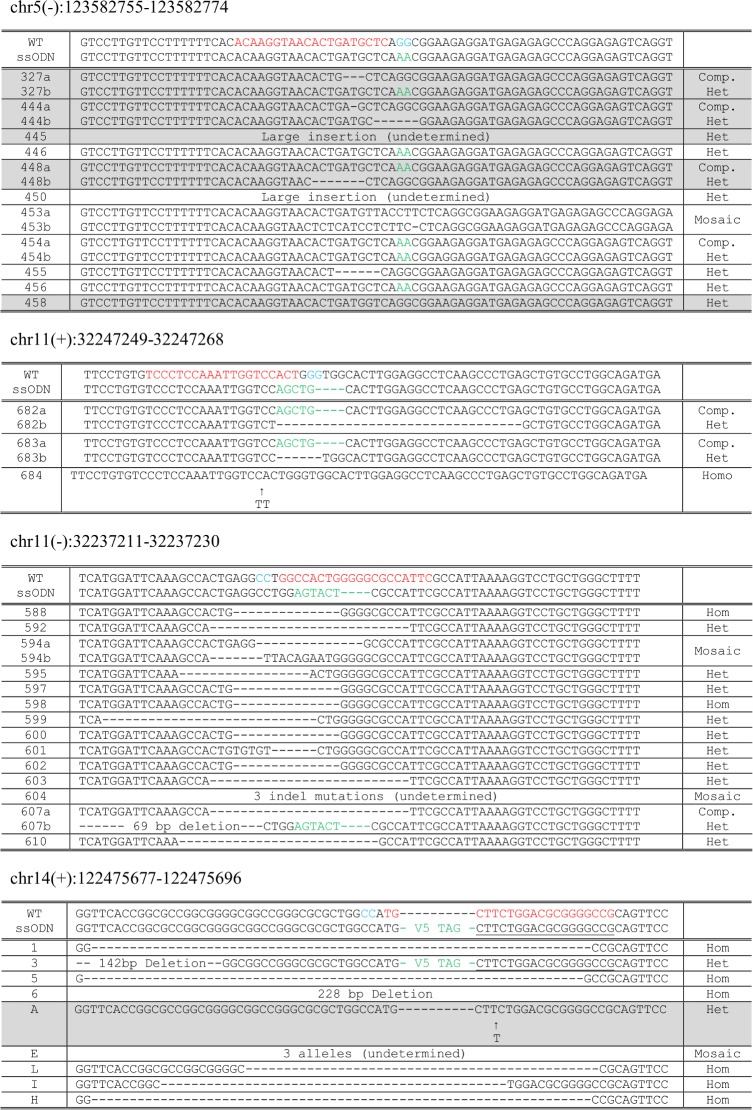
Alleles identified in mutant pups generated from microinjection of guide-RNA and ssODN into fertilized zygotes derived from Cas9 expressing female mice. Alleles identified in each mutant founder mice are aligned against the wild-type sequence. In homozygous mutant mice, only one allele is shown. In heterozygous alleles with an intact wild-type allele present, only the mutant allele is shown. In compound heterozygous mutant mice with two mutant alleles, both alleles are shown. Where more than two alleles, in addition to the wild-type sequence are present (mosaic founders), the mutant alleles identified are shown. Protospacers in the wild-type sequence are shown in red, with the PAM motif highlighted in blue. For HDR ssODN templates the variant nucleotides are shown in green. Where the mutated offspring had not inherited the Cas9 transgene, the entry in the table is marked with a grey background.

Having established in whole zygotes that a genetic Cas9 supply led to an increase in the efficiency of HDR using an ssODN template, we sought to test the methodology for the production of live point mutation knock-in mice at the locus previous studied and at three further genomic loci (Table B in S1 File). Zygotes prepared from Cas9 overexpressing mothers were microinjected with sgRNA and ssODN (Table D in S1 File), cultured overnight, transferred to foster mothers and resulting offspring were genotyped at the target loci by PCR amplification and Sanger sequencing. Table 2 summarizes the results and the predominant alleles obtained in the resulting pups are shown in detail in Fig 6. Efficient generation of mutant pups harbouring the desired knock-in allele was observed for two loci (29% and 50%), confirming that genetic supply of Cas9 is a viable strategy for the generation of live knock-in mice using ssODN donor templates. Founder mice harbouring these knock-in alleles were able to transmit the mutations to the next generation, again confirming that genetic supply of Cas9 is suitable for the generation of lines of site-specific modified mice. The other two projects yielded a lower rate of knock-in (6% and 4%) and these alleles suffered additional non-specific indels, presumably due to either reprocessing of the recombined allele by further CRISPR/Cas9 action or through aberrant repair, as has been observed in other studies using ssODN[28, 29].

**Table 2:** Production data for knock-in mouse models generated using donor Cas9 expressing females.

Genomic targets (protospacers)	Injection mix	Genotype of oocyte donor	Number of embryos transferred	% of pups born per embryos transferred	Number of pups / transferred embryos (%)	Mutant pups / analyzed pups (%)
chr5(-): 123582755–123582774	20 ng/ul sgRNA	Heterozygous	110	21%	11/17 (65%)*	Yes
	20 ng/ul ssODN				5/19 (29%) HDR*	
chr11(+): 32247249–32247268	20 ng/ul sgRNA	Homozygous	60	13%	3/4 (75%) mutated	Yes
	20 ng/ul ssODN				2/4 (50%) HDR	
chr11(-): 32237211–32237230	20 ng/ul sgRNA	Homozygous	50	48%	14/24 (58%)	N/A
	20 ng/ul ssODN				1/24 (4%) HDR (imperfect)	
chr14(+): 122475677–122475696	20 ng/ul sgRNA	Heterozygous	44	41%	9/18 (50%)*	N/A
	20 ng/ul ssODN				1/18 (6%) HDR (imperfect)	

* Mutant mice were identified which were negative for the Cas9 transgene (see Fig 6).

### Maternal Cas9 contribution for the generation of mutant pups and analysis of off-target site mutagenesis

Consistent with the observation that all zygotes derived from Cas9 expressing mothers have maternal Cas9 present, irrespective of the inheritance of the Cas9 transgene, we explored whether this maternally contributed Cas9 was suitable for the generation of live pups. We thus genotyped the founder pups generated from the microinjection experiments that used heterozygous females as embryo donors and found many of the founders had indeed not inherited the Cas9 transgene (Figs 5 and 6). This was the case for founder mice harbouring both indel and knock-in alleles and thus confirmed that Cas9, supplied as maternal transcript or protein, in the absence of transgene inheritance, is sufficient for the generation of live indel and point mutation knock-in mice.

For the mutant mice that had inherited the Cas9 transgene, persistent expression of Cas9 in the resulting mice might be expected to increase the likelihood of off-target cleavage, as has been reported for the prolonged expression of CRISPR/Cas9 nucleases in vitro[30]. We examined the top six off-target sites, predicted by the mit.crispr.edu algorithm, in three mutant founder mice, which were heterozygous for the CAG-Cas9 transgene and found no evidence for off-target mutation by Sanger sequencing (Table F in S1 File).

### Use of cryopreserved zygotes from Cas9 overexpressing mice for microinjection

**Fig 7:**
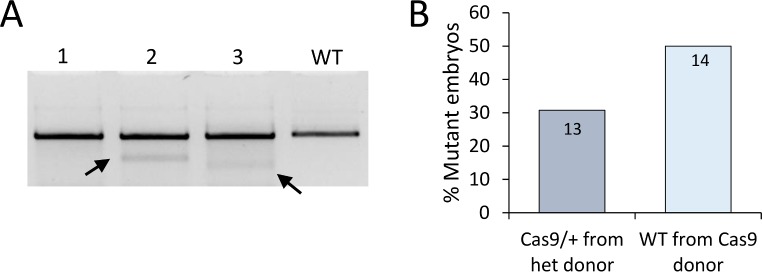
Mutagenesis with cryopreserved embryos and using synthetic crRNA:tracrRNA. (A) Target site amplification in blastocysts after microinjection of thawed zygotes with sgRNA. Zygotes had previously been prepared from heterozygous Cas9 expressing females and cryopreserved by vitrification. Doublets are indicative of indel mutagenesis (black arrows). (B) Percentage mutation rates (mutant embryos per total number) in either heterozygous (Cas9/+) or wild-type (WT) embryos obtained from heterozygous Cas9 expressing females, following microinjection with synthetic crRNA:tracrRNA alone. Data inside the bars are the number of embryos sampled.

One disadvantage of using genetically supplied Cas9 is the need to maintain and breed a line of Cas9 overexpressing mice. Frequently, facilities generating genetically manipulated mice rely on commercial suppliers to provide their donor female mice for embryo production as it can be more cost-effective than an in-house breeding program for donor mice. In a small pilot study, we examined the use of cryopreserved zygotes, derived from Cas9 overexpressing mice, to establish whether a frozen resource could be used instead of freshly prepared zygotes from live colonies. Zygotes were prepared from heterozygous Cas9 female mice and cryopreserved by vitrification[31]. Upon thawing, zygotes were microinjected with sgRNA against target chr17(+):34030557–34030576 and cultured to the blastocyst stage. Clear evidence of site-specific mutagenesis at the target site was observed by polymorphic amplification products, obtained from whole blastocysts, analysed on a high percentage agarose gel and confirmed by Sanger sequencing (Fig 7A).

### Microinjection of zygotes prepared from Cas9 overexpressing mice with synthetic tracrRNA and crRNA

As an alternative to sgRNA, the two component RNA molecules of the native CRISPR/Cas9 system (the invariant tracrRNA and the crRNA specific for the target gene), can be used to address the Cas9 cleavage. Microinjection of this 3-component CRISPR/Cas9 system in mice has been shown to yield good rates of site-specific transgene insertion[19]. The tracrRNA and crRNA components are of a size that can be readily synthesized and subsequently their use avoids the need to generate high quality sgRNA by in vitro transcription. We synthesized a 69mer tracrRNA and a 42mer crRNA against the protospacer chr17(+):34030557–34030576 and microinjected these into zygotes derived from Cas9 expressing mice. Following culture to the blastocyst stage, embryos were genotyped and 41% (11/27) were found to have significant levels of mutagenesis at the target locus, and mutant embryos were recovered which had not inherited the Cas9 transgene, consistent with maternal supply (Fig 7B).

## Discussion

We have explored the genetic supply of the nuclease Cas9 using a transgenic model which harbours a single copy of the Cas9 overexpression construct within the *Gt(ROSA26)Sor* locus to facilitate mouse model generation using the CRISPR/Cas9 system. Genetic supply of Cas9 to zygotes had previously been shown to mediate site-specific mutagenesis in two studies that generated Cas9 expressing transgenic mice by pronuclear microinjection of a Cas9 expression construct under the control of the CAG promoter[16] and the oocyte specific ZP3 promoter[17]. These studies showed mutagenesis of target loci by indel mutagenesis in embryos and live mice, respectively. We have extended these ideas and, using our targeted transgenic Cas9 expressing mouse, present a side-of-side comparison of mutagenesis efficiencies using different sources of Cas9 for both whole embryo analysis and for mouse model generation and show for the first time that this strategy can be used to generate knock-in mice lines using ssODN as template donors.

Although we also observed no statistical difference in mutation rate when analysing whole embryos using 2 different sgRNAs, when using genetic supply for live model generation, we did see a consistent increase in efficiency when compared with exogenous supply by mRNA or protein microinjection across multiple genomic loci. Our analysis of embryos did, however, highlight that the outcome of investigations into CRISPR/Cas9 efficiencies are extremely dependent upon the genomic locus or the sgRNA used, as a very significant increase in mutational efficacy (see below) observed for genetic supply compared with exogenous supply did not hold up at an alternative loci. For methodological improvements, it is thus essential to test them at a variety of different genomic loci before broad conclusions can be drawn.

The two previous studies of transgenic Cas9 mice made by a random integration approach used two different promoters. The same CAG promoter used in our study was chosen by Sakurai and colleagues[16] who compared mutagenesis rates using genetic Cas9 supply to exogenous supply as mRNA for one loci and reported no significance difference in mutagenesis rates at the blastocyst stage, consistent with our results. This report did not use the transgenic model for the generation of live founder mice. An oocyte specific promoter (ZP3) was chosen by Zhang and colleagues[17] for the purpose of model generation and strikingly reported a lower efficiency for genetic supply when compared to exogenous supply by mRNA microinjection (side by side comparison was performed for a single sgRNA/locus). It is interesting to speculate that the strength of the ZP3 promoter may be lower than the CAG promoter, which is known to drive very strong transgenic expression[32], and this might explain this lower rate of mutagenesis seen with this mouse. It is, however, difficult to speculate on absolute expression level as both these studies used a random integration approach and thus the expression level would be very dependent upon the particular transgenic founder line used for zygote production and could show variegation[33]. Furthermore, both studies only performed quantitative analysis at a single genomic target site.

Maternal-effect genes were described in mammals some years ago (reviewed by [34]) and it is well established that oocytes are loaded with maternal factors encoded by maternal genes that accumulate during oogenesis. Prior to the activation of the embryonic genome after the first cleavage event, the genotype of the mother thus has a considerable influence during preimplantation development. In agreement, we found that the insertion of only a single copy of the CAG-Cas9 transgene into the *Gt(ROSA26)Sor* locus produced oocytes with sufficient maternal Cas9 activity to target the genome efficiently. Furthermore, we found that the zygosity of the embryo donor used did not influence mutagenesis rate nor the extent of mutagenesis within each individual embryo, perhaps indicating that maternal Cas9 from heterozygous mothers was already at saturation levels within the zygote. These maternally contributed tools are particularly advantageous with respect to mouse model production as wild-type backcrossing to remove the transgene can be avoided by selecting genetically modified offspring which have not inherited the transgene.

When injecting sgRNA chr17(+):34030557–34030576, embryos receiving a maternal supply of Cas9 had a much larger and more consistent degree of mutagenesis when compared with exogenous supply of Cas9 by mRNA microinjection. It is possible that upon injection of the sgRNA, the active Cas9 can be recruited more rapidly to its genomic target site if the Cas9 nuclease is already present as maternal protein within the fertilized zygote. In contrast, when supplying Cas9 as mRNA, the recruitment of an active nuclease would be expected to be slower as translation and transport of Cas9 into the nucleus must first occur. It is interesting that maternal supply was also more mutagenic than Cas9 supply as exogenous protein, since this would also be expected to be active immediately. Potentially the local concentration of Cas9 when delivered as maternal protein is higher than that achievable by exogenous microinjection. When using a different sgRNA addressing a different genomic location, no difference in mutagenic efficiency was observed–potentially this genomic site is less amenable to nuclease cleavage so the advantages of early and immediate activity are lost.

An additional advantage for a transient and immediate activity of Cas9 has been discussed in the context of whether HDR or NHEJ is employed as the preferred repair mechanism. It is clear that NHEJ repair occurs at a much higher frequency that HDR and finding ways of encouraging HDR or inhibiting NHEJ is currently an active field of research to maximize the application of the CRISPR/Cas9 system. With respect to zygote microinjection, initial reports concerning the application of NHEJ inhibitors are encouraging[35] but it remains to be seen whether this kind of pharmacological intervention is suitably robust for all situations. A simpler approach might be to consider that a faster nuclease action may increase the chances of HDR events since the donor DNA template, frequently a linear piece of single stranded or double stranded DNA, is quickly degraded by cellular exonucleases. Confirming the fast-is-better hypothesis, previous reports have shown that, in some contexts, microinjection of Cas9 protein when compared with mRNA tend to achieve higher rates of HDR[19–21]. It is thus of interest that we found an increase in knock-in efficiency when using genetic supply of Cas9 as compared with Cas9 mRNA. As zygotes derived from Cas9 expressing females are pre-loaded with maternal Cas9, it seems plausible that the high rates of HDR reported in this work could be related to a faster nuclease cleavage. Of particular note is that knock-in efficiency when Cas9 was delivered genetically was also found to be higher than Cas9 supply as microinjected protein; as discussed above it could be that the absolute concentrations achievable by exogenous supply are less that maternal supply. Future work will address whether genetic supply achieves higher rates of HDR versus conventional exogenous supply across other genomic loci.

The maternal supply of Cas9 might provide an elegant way of restricting a pulse of CRISPR/Cas9 activity to the fertilized zygote prior to the first cleavage event. Restricting the activity of CRISPR/Cas9 to the one-cell embryo could theoretically reduce the mosaicism and allele complexity that is frequently seen following microinjection experiments [13, 36]. Interestingly, we failed to detect any correlation between the source of Cas9 (genetic versus protein or mRNA supply) and the allele complexity when comparing whole embryo genotypes using two genomic target sites. Despite some mosaicism, analysis of the offspring derived from tested founders produced by maternal supply of Cas9, confirmed germline transmission of the desired mutation in all cases. Sakurai et al.[16], using essentially the same promoter element to drive Cas9 expression as in this study, observed alterations in allele complexity but also reported no statistically significant effects. Interestingly, the study which restricted Cas9 activity to the oocyte by driving its expression under the control of the ZP3 promoter[17], did demonstrate a significant reduction in allelic complexity when using zygotes genetically supplied with Cas9 in this manner. Potentially the time-restricted, oocyte-specific expression of Cas9 contributed to this reduced mosaicism, but the general lower level of mutagenic activity reported in this line may also provide an explanation for this observation.

From a practical standpoint, genetic supply using zygotes from Cas9 expressing mice lines is advantageous as zygotes receive a consistent dose of Cas9 and no Cas9 mRNA or protein need be generated, purchased and quality-controlled. Furthermore, any difficulties arising from batch-to-batch variability in commercial preparations of Cas9 protein and mRNA would be avoided by using a genetic source. This may be particularly useful for laboratories establishing CRISPR/Cas9 microinjection techniques for the first time. We have also shown that efficient mutagenesis can be achieved using synthetic preparations of tracrRNA and crRNA, in combination with genetic supply of Cas9 –thus all in-house RNA synthesis can be avoided which may be an attractive option for core facilities without extensive molecular biology resources. Lastly, we have shown in a small pilot study that cryopreserved zygotes can be microinjected with sgRNA to achieve site-specific mutagenesis, meaning that, once an archive of cryopreserved embryos is generated, the maintenance of a Cas9 overexpressing colony would not be necessary. One clear disadvantage of this methodology, is that it cannot be applied on different strains of mice, in contrast with exogenous supply of Cas9 which has the flexibility to be used on potentially any genetic background, such as disease model strains[37].

Recently other routes for the introduction of CRISPR/Cas9 components into reproductive tissues have been reported. Instead of microinjection, zygote electroporation can be used to introduce the active nuclease into the embryo[38, 39]. Alternatively, direct *in vivo* electroporation of reproductive tissues with CRISPR/Cas9 nucleases have also been shown to be a successful strategy[40]. All of these new techniques would be expected to be entirely compatible when applied on a transgenic mouse overexpressing Cas9. Indeed, the genetic supply could potentially make the method more efficient as only sgRNA and template for HDR need be introduced.

In conclusion, the maternal supply of Cas9 to fertilized oocytes provides a further improvement for site-specific mutagenesis in the mouse using CRISPR/Cas9 nucleases. Firstly, the production of deletion and point mutation knock-in mouse model reported here using maternally supplied Cas9 suggests that genetic supply can provide a robust alternative to exogenous supply and avoids the variability inherent in sourcing or generating and delivering Cas9 mRNA or protein. Secondly, at certain genomic sites, maternal supply of Cas9 may actually allow higher levels of mutagenesis and increased knock-in efficiencies. Lastly, the use of heterozygous donor females for maternal contribution of Cas9 ensures the efficient generation of founder mice without the inheritance of the Cas9 transgene.

## Supporting Information

S1 File **Table A.** Protospacer information, sgRNA cloning oligos and genotyping primers for in vitro validation of Cas9 overexpressing mouse model. **Table B.** Genomic target site, sgRNA cloning oligos, primers for in vitro transcription template amplification and genotyping primers for in vivo experiments. **Table C.** Genotyping primers for Cas9 transgene. **Table D.** ssODN sequences used for knock-in experiments. **Table E.** Microinjection production data. **Table F.** Genotyping primers used for analysis of off-target mutagenesis.
(DOCX)
