# Supplementary material for: Maternal Supply of Cas9 to Zygotes Facilitates the Efficient Generation of Site-Specific Mutant Mouse Models
Source: PLoS One. 2017 Jan 12;12(1):e0169887. doi: 10.1371/journal.pone.0169887 (PMC5231326; doi:10.1371/journal.pone.0169887)
Supplement: S1 File — Table A. Protospacer information, sgRNA cloning oligos and genotyping primers for in vitro validation of Cas9 overexpressing mouse model. Table B. Genomic target site, sgRNA cloning oligos, primers for in vitro transcription template amplification and genotyping primers for in vivo experiments. Table C. Genotyping primers for Cas9 transgene. Table D. ssODN sequences used for knock-in experiments. Table E. Microinjection production data. Table F. Genotyping primers used for analysis of off-target mutagenesis. (DOCX) [file pone.0169887.s001.docx]

**Table A**

| Locus | Protospacer / PAM | guide-RNA cloning oligos | Genotyping primers |
| --- | --- | --- | --- |
| *Trp53* | GGACGATCTGTTGCTGCCCC AGG | TCCGGACGATCTGTTGCTGCCCC | GGGGGACTGCAGGGTCTCAGAAGTT |
|  |  | AAACGGGGCAGCAACAGATCGTC | AGACTTGGCTGTCCCAGACTGCAG |
| *Cdkn2a* | GCAGGTTCTTGGTCACTGTG AGG | TCCGCAGGTTCTTGGTCACTGTG | CAGCTTCGGAGGGCCTTTCCTACCT |
|  |  | AAACCACAGTGACCAAGAACCTG | CTCGAGGTGCCTCAACGCCGAAGG |

**Table B**

| Genomic target site | guide-RNA cloning oligos | PCR primers for amplification of the T7  in vitro transcription template | Genotyping primers |
| --- | --- | --- | --- |
| chr17(+):34030557-34030576 | CACCGTTACTTACCCAAGGCATGC | TTAATACGACTCACTATAGGTTACTTACCCAAGGCATGC | GTTCTTAATCGGTGGGAAGCTC |
|  | AAACGCATGCCTTGGGTAAGTAAC | AAAAGCACCGACTCGGTGCC | CAGCACACCAGTCCCTGGTTTT |
| chr10(-):41451292-41451311 | CACCGCATTGAGAAATACTCGCTAC | AGTCCTTAATACGACTCACTATAGGCATTGAGAAATACTCGCTAC | AAGCCTCTTCAGAGTCTTCTGG |
|  | AAACGTAGCGAGTATTTCTCAATGC | AAAAGCACCGACTCGGTGCC | GCGTATGCCTTTACCAGGTCAT |
| chr11(-):32237211-32237230 | CACCGAATGGCGCCCCCAGTGGCC | TTAATACGACTCACTATAGGAATGGCGCCCCCAGTGGCC | ACAGCAACCATCTGGGTGAG |
|  | AAACGGCCACTGGGGGCGCCATTC | AAAAGCACCGACTCGGTGCC | TGCTGGTGTCTGTGGACAAG |
| chr11(+):32247249-32247268 | CACCGTCCCTCCAAATTGGTCCACT | TTAATACGACTCACTATAGGTCCCTCCAAATTGGTCCACT | AGCCCACAACTTCCTGTCTT |
|  | AAACAGTGGACCAATTTGGAGGGAC | AAAAGCACCGACTCGGTGCC | GCTGGCCTGGAACTAACTCA |
| chr5(+):75961998-75962017 | CACCGCCTATACACAACAGTGCGA | TTAATACGACTCACTATAGGCCTATACACAACAGTGCGA | TTTGACTTCTCTCTCGTTCAGGAGTGCC |
|  | AAACTCGCACTGTTGTGTATAGGC | AAAAGCACCGACTCGGTGCC |  |
| chr5(-):75962425-75962444 | CACCGACACTGCTCCTCCTTATCTG | TTAATACGACTCACTATAGGACACTGCTCCTCCTTATCTG | TAGCCCTGGTGGATTCCTCTCC |
|  | AAACCAGATAAGGAGGAGCAGTGTC | AAAAGCACCGACTCGGTGCC |  |
| chrX(-):41620875-41620894 | CACCGCCACAGTCAGGAAAGCAGCA | TTAATACGACTCACTATAGGCCACAGTCAGGAAAGCAGCA | AACTAGAGAAAACCTGGAGAGGCC |
|  | AAACTGCTGCTTTCCTGACTGTGGC | AAAAGCACCGACTCGGTGCC | CCCGTGAGTCTAATGGACAATGGA |
| chr14(+):122475677-122475696 | CACCGCGGCCCCGCGTCCAGAAGCA | AGTCCATTAATACGACTCACTATAGGCGGCCCCGCGTCCAGAAGCA | CTGGCTTTGGACTCTTCTCCTCCT |
|  | AAACTGCTTCTGGACGCGGGGCCGC | AAAAGCACCGACTCGGTGCC | ATTTGCCCATTGAGCACGTTCTGC |
| chr5(-):123582755-123582774 | CACCGACAAGGTAACACTGATGCTC | AGTCCATTAATACGACTCACTATAGGGACAAGGTAACACTGATGCTC | GGAGATGCTAGTACCCCTAGGTGGC |
|  | AAACGAGCATCAGTGTTACCTTGTG | AAAAGCACCGACTCGGTGCC | ATGTCTGCAACAGGTGAGCCCC |

**Table C**

| PCR target | Genotyping primers |
| --- | --- |
| Cas9 expression cassette integrated at the *Gt(ROSA26)Sor* locus | GCACTAGTTCTAGAGCGATCCCC |
|  | CGGGAGAAATGGATATGAAGTACTGGGC |
| Control gene *(Zbtb24)* | AAGCCTCTTCAGAGTCTTCTGG |
|  | GCGTATGCCTTTACCAGGTCAT |

**Table D**

| Genomic target site | HDR template (ssODN) |
| --- | --- |
| chr11(-):32237211-32237230 | TAGTGAGATCTGGCCTCATGGATTCAAAGCCACTGAGGCCTGGAGTACTCGCCATTCGCCATTAAAAGGTCCTGCTGGGCTTTTCTAGCTCCAGATTCCAGATTTTTGGCAGCCACTGGTACTTACAGACACACA |
| chr11(+):32247249-32247268 | TGTCATCTGCCAGGCACAGCTCAGGGCTTGAGGCCTCCAAGTGCagcTGGACCAATTTGGAGGGACACAGGAATTTGAGCTTTTGGTGAAAAGGCATTCAAGGTCCTACTAGCCAGATACCCTGTTTGTTAGTGG |
| chr14(+):122475677-122475696 | ggcggcggggttcaccggcgccggcggggcggccgggcgcgctggccatgGGCAAGCCTATCCCTAACCCTCTGCTGGGCCTGGACAGCACCcttctggacgcggggccgcagttcccggccatcggggtgggcagcttcgc |
| chr5(-):123582755-123582774 | GATGTGTTTCCCAGTGCTCCCTCTGATGTCCTTGTTCCTTTTTTCACACAAGGTAACACTGATGCTCAAACGGAAGAGGATGAGAGAGCCCAGGAGAGTCAGGTAATGCTCTTCTATTACCTTCTGCTGTCTGCATGCC |

**Table E**

|  | No. of embryos injected | No. of survived embryos | % lysis rate | No. of 2 cell embryos | % 2 cell progression | Embryos Transferred | Pups born | % Live birth |
| --- | --- | --- | --- | --- | --- | --- | --- | --- |
| Cas9  (Hom and Het) | 1753 | 1539 | 12.21 | 976 | 63.4 | 675 | 160 | 23.7 |
| Wild-type | 2614 | 2246 | 14.08 | 1491 | 66.4 | 1097 | 213 | 19.4 |

**Table F**

| Off-target site | Genotyping primers |
| --- | --- |
| CCACAGGCAGGAGAGCAGCA AGG  chr9(-):22830549-22830571 | GCCAGTTTTCTGACCTTGTGC |
|  | GCAAGAAACAGAATCCCGGTC |
| AGACAGGCAGGAAAGCAGCA GGG  chr2(+):101568152-101568174 | GGTGCGGCATTTCAAGTCTG |
|  | TCCCAGTTGTTGCTCACCTC |
| AAACAGTCGGGAAAGCAGCA GGG  chr8(-):49562872-49562894 | GCAGAAGGACAATCGAGGGG |
|  | CCCCTCCCTCAAAGTCGGAT |
| GCAGAGTCTGGAAAGCAGCA GGG  chr8(+):44900060-44900079 | GCCGGCAGCTGTATTAGGAA |
|  | GCTGTTGGGTGTTCTTCTGC |
| CCACAGGGATGAAAGCAGCA AGG  chr15(+):48409469-48409491 | GGGAAAGGATGTCTGAGATGGT |
|  | GGTGAAGTGTCGCTTGCTGT |
| CCAAAGTTAGAAAAGCAGCA AGG  chr19(-):56096240-56096262 | CACACTTTCGCCTGGACAAC |
|  | CAAGTTTGAATCCGACGTGGT |
